# Hot Deformation Behavior and Microstructure Evolution of a Graphene/Copper Composite

**DOI:** 10.3390/ma17164010

**Published:** 2024-08-12

**Authors:** Tiejun Li, Ruiyu Lu, Yuankui Cao, Bicheng Liu, Ao Fu, Bin Liu

**Affiliations:** State Key Laboratory of Powder Metallurgy, Central South University, Changsha 410083, China; litj1990@163.com (T.L.); 233302064@csu.edu.cn (R.L.); 18584439317@163.com (B.L.); aofu_ice@csu.edu.cn (A.F.)

**Keywords:** graphene/copper composite, hot deformation behavior, dynamic recrystallization, microstructure

## Abstract

Graphene/copper composites are promising in electronic and energy fields due to their superior conductivity, but microstructure control during thermal mechanical processing (TMP) remains a crucial issue for the manufacturing of high-performance graphene/copper composites. In this study, the hot deformation behavior of graphene/copper composites was investigated by isothermal compression tests at deformation temperatures of 700~850 °C and strain rates of 0.01~10 s^−1^, and a constitutive equation based on the Arrhenius model and hot processing map was established. Results demonstrate that the deformation mechanism of the graphene/copper composites mainly involves dynamic recrystallization (DRX), and such DRX-mediated deformation behavior can be accurately described by the established Arrhenius model. In addition, it was found that the strain rate has a stronger impact on the DRX grain size than the deformation temperature. The optimum deformation temperature and strain rate were determined to be 800 °C and 1 s^−1^, respectively, with which a uniform microstructure with fine grains can be obtained.

## 1. Introduction

Copper-based materials are extensively used in electric power and rail transportation such as electrical contacts for vacuum switches and conductive cables for power transmission, due to their prosperous electrical properties [1,2]. However, their relatively poor mechanical properties greatly limit their further uses [3]. Introducing a reinforcing phase to manufacture composites is the most effective strategy for achieving excellent strength. Among current reinforcing phases, graphene is considered a perfect reinforcing phase because of its outstanding mechanical properties (130 GPa tensile strength) and high electrical properties (2 × 10^5^ cm^2^·V^−1^·S^−1^ carrier mobility) [4]. Cao et al. [5] showed that 1.6 vol.% of graphene can increase the tensile strength from 218 MPa to 305 MPa, maintaining 97.1% IACS high electrical conductivity at the same time. In graphene/copper composites, graphene always forms a three-dimensional continuous network structure through powder metallurgy, leading to load-transfer strengthening [6,7]. However, the weak graphene/copper interfacial bonding makes the composites prone to cracking under loads, resulting in increased brittleness [8,9,10].

Thermal mechanical processing (TMP) was considered an effective way to modify the interfacial bonding between copper and graphene, simultaneously producing strong load-transfer strengthening and grain boundary strengthening [11,12]. Li et al. [13] increased the tensile strength of graphene/copper composites from 176 MPa to 573 MPa by hot extrusion while maintaining excellent conductivity and moderate plasticity. Que et al. [14] also demonstrated that the tensile strength of composites can be increased to 500 MPa by hot rolling while displaying a conductivity value of 94.2% IACS and a plasticity value of 20%. These studies provide effective guidance for improving the comprehensive performance of the alloy, but TMP parameters such as deformation temperature should be carefully chosen to stabilize the plastic deformation and avoid defects. Xiu et al. showed that when the deformation temperature was lower than 500 °C, cracks were generated inside the composite material, reducing the densification and hardness [15]. Yang et al. found that the exorbitant deformation temperature decreased the mechanical properties of composites significantly [16]. At present, the mechanism of TMP parameters influencing the composite’s microstructural evolution is still unclear. More investigation into the relationship between hot deformation behavior and TMP parameters should be conducted.

In this investigation, the flow behavior of graphene/copper composites at different TMP parameters was investigated. The constitutive model and hot processing map were established, and the deformation mechanism of the composites was discussed. The TMP parameters of such composites are optimized to offer a theoretical basis for manufacturing high performance graphene/copper composites.

## 2. Materials and Methods

The manufacturing method of graphene/copper powders used in this study is consistent with previous reports [13]. The above powders were sintered at 900 °C for 10 min under a sintering pressure of 50 MPa by rapid hot press sintering equipment (FHP-828), with a temperature increase rate of 100 °C/min. Isothermal compression tests were performed by Gleeble-1500 with a temperature of 700~850 °C and a strain rate of 0.01~10 s^−1^. The blank was Φ8 × 12 mm, and the vacuum was always below 10 Pa during the experiment. The heating rate is 5 °C/s. The temperature of the samples was held for 2 min after researching the setting temperature. Samples were deformed by 50% and rapidly quenched when the deformation was completed.

The micro-morphology of the composites before and after deformation was analyzed by a field emission scanning electron microscope (Quanta-FEG250, FEI Company in Hillsboro, OH, USA) equipped with an EBSD detector (AZtec EBSD, London, UK). The observed cross-section is in the core of specimens and is parallel to the deformation direction. Samples were electrolytically polished to remove the surface stress layer of samples. The electrolyte was 70 vol% phosphoric acid alcohol solution with a voltage of 5 V and a time of 10~20 s.

## 3. Results and Discussion

### 3.1. Microstructure of Graphene/Copper Powders and As-Sintered Composites

**Figure 1:**
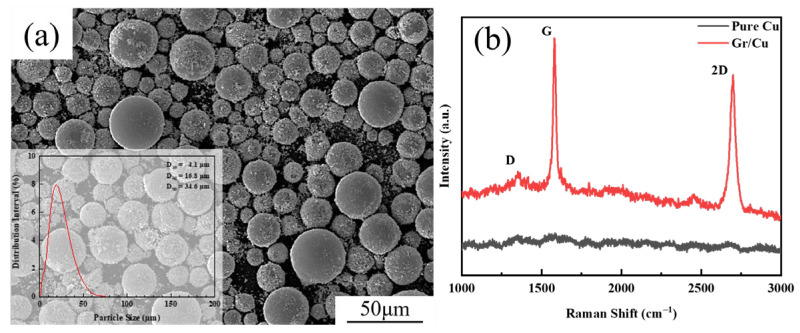
Graphene/copper powders: (**a**) powder characteristics; (**b**) Raman spectroscopy.

Figure 1a reveals the surface morphology and the particle size distribution of composite powders. Composite powders are mainly micron-sized spherical powders, and a small amount of satellite powders are also attached to the surface of the micron powders. The particle size distributes 0.7~66.9 μm, and the D_50_ is 16.8 μm. Figure 1b shows the Raman spectrum of graphene. Three characteristic peaks of graphene can be observed: a D peak with weak intensity located at around 1340 cm^−1^, a sharp G peak lying at around 1580 cm^−1^, and a sharp 2D peak at around 2700 cm^−1^. In the Raman spectrum of graphene, the D peak is due to the breathing mode of defects such as hybridized sp^3^. The G peak is due to the in-face vibration of the hybridized sp^2^ in graphene. The 2D peak is caused by the electronic energy band structure of graphene, which can respond to the number of graphene layers [17,18]. The defect density of graphene is directly proportional to the intensity ratio of the D peak to the G peak (I_D_/I_G_). The number of graphene layers is inversely proportional to the intensity ratio of the 2D peak to the G peak (I_2D_/I_G_) [19,20]. After calculation, the value of I_D_/I_G_ is 0.14, which proves that the defect concentration of graphene in the composite powder is very low. The value of I_2D_/I_G_ is 0.89, which proves that the number of graphene layers are very few.

**Figure 2:**
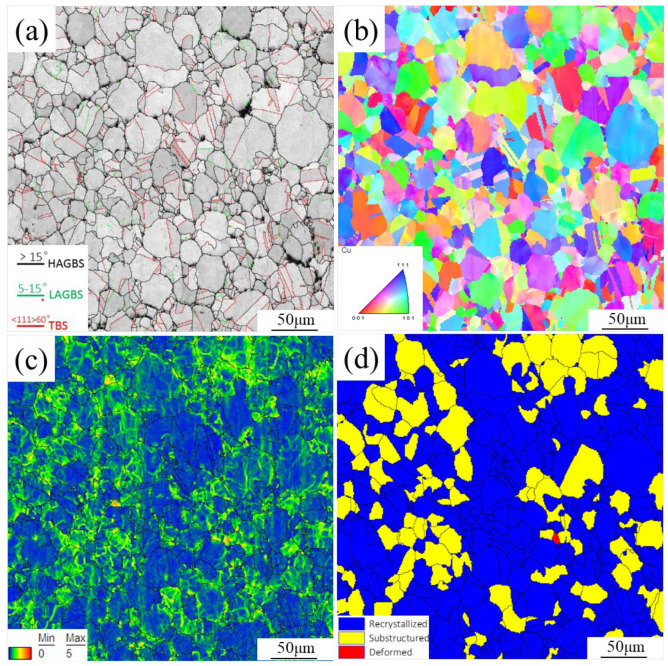
EBSD structure of graphene/copper composites sintered at 900 °C: (**a**) IQ map; (**b**) IPF map; (**c**) KAM map; (**d**) recrystallization map.

EBSD microstructures of as-sintered composites are shown in Figure 2. The IQ (image quality) map shows the distribution of high angle grain boundaries (HAGBS), low angle grain boundaries (LAGBS), and twin boundaries (TBS) in composites, represented by black, green, and red, respectively. The grain boundaries of composites are mainly composed of TBS and HAGBS. The IPF (inverse pole figure) map shows grain orientations in composites, with each crystal corresponding to one of the colors in the triangle color card. The grain orientation of composites is random. Grains are basically spherical with an average size of 22.1 μm, which is similar to the D_50_ of powders, proving that graphene can prevent grain growth during the sintering process [21]. The KAM (kernel average misorientation) map shows the local misorientation of composites, where small and large misorientation are shown in blue and green, respectively. A high orientation difference at grain boundaries indicates a high dislocation density at the graphene/copper interface. Figure 2d shows the recrystallization map, where the recrystallized grains, substructured grains, and deformed grains are indicated in blue, yellow, and red, respectively. The percentage of recrystallized grains is 60.9%, while the remaining grains are almost not deformed.

**Figure 3:**
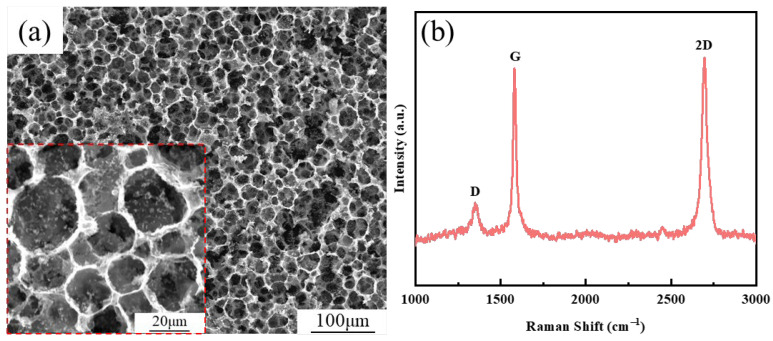
Distribution of graphene in as-sintered composites: (**a**) low magnification; (**b**) high magnification. The inset shows the Raman spectrum of graphene.

To observe the spatial distribution of graphene in composites, the graphene/copper composites were put into an aqueous ferric chloride hydrochloric acid solution for immersion corrosion. As shown in Figure 3a, the network of graphene was observed, and the diameter of the mesh was the same as that of composite powders. Figure 3b reveals the Raman spectrum of graphene in composites, and three characteristic peaks of graphene can still be observed. After calculation, the value of I_D_/I_G_ is 0.22, proving that the defect concentration of graphene in the composite powder is still very low. The value of I_2D_/I_G_ is 1.04, which proves that the number of layers of graphene is low.

### 3.2. Constitutive Equation and Hot Processing Map

**Figure 4:**
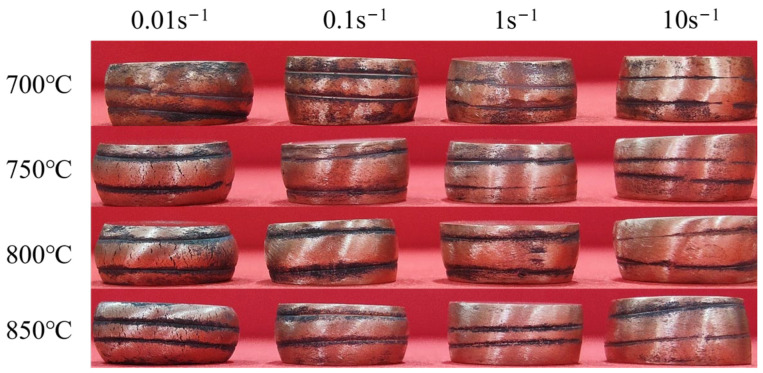
Macrophotographs of graphene/copper composites after hot deformation (black parts on the surface of the sample are oxidized skins that have not been completely cleaned off after hot deformation).

**Figure 5:**
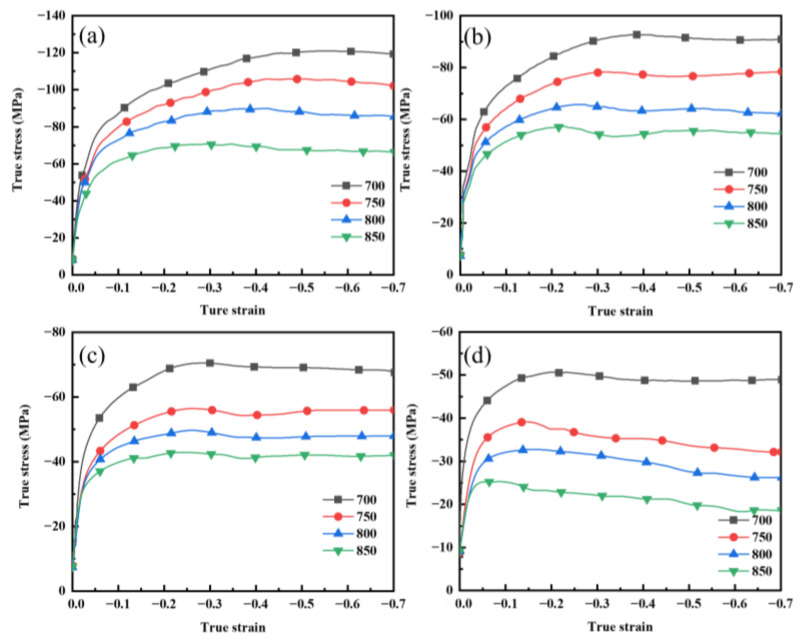
True stress–strain curves of graphene/copper composites in different deformation conditions: (**a**) $\dot{\varepsilon }$ = 10 s^−1^; (**b**) $\dot{\varepsilon }$ = 1 s^−1^; (**c**) $\dot{\varepsilon }$ = 0.1 s^−1^; (**d**) $\dot{\varepsilon }$ = 0.01 s^−1^.

Figure 4 shows macroscopic photographs of Graphene/Cu composites deformed 50% at different TMP parameters (700~850 °C and 0.01~10 s^−1^), from which no large crack is observed in all samples, but the deformation of samples with higher strain rates was not uniform. True stress–strain curves are shown in Figure 5. At high deformation temperature and low deformation rate, the flow stress increases rapidly at first and slowly decreases to a steady stage subsequently, which is a typical feature of dynamic recrystallization [22,23]. At the early stage of deformation, work-hardening dominates, and the softening caused by dynamic recovery is not enough to counteract work-hardening. With the increase in strain, dislocations gradually proliferate and entangle with each other, so the flow stress increases rapidly. While dislocations proliferate to a threshold, dynamic recrystallization is triggered, and dislocations begin to climb, rearrange, and counteract each other. Thus, the flow stress decreases slowly. When work-hardening and dynamic recrystallization softening reach dynamic balance, the flow stress changes lightly and enters into a steady flow stage [23,24].

As the deformation temperature decreases and the strain rate increases, the peak stress gradually increases, and the strain required to transfer into the steady flow stage also gradually increases. If the deformation temperature is 800 °C, when the strain rate is increased from 0.01 s^−1^ to 10 s^−1^, the peak flow stress is increased from 33 MPa to 90 MPa, and the true strain entered into the steady flow stage is also increased from 0.15 to 0.38. Comparing flow stress curves of composites (a deformation temperature of 800 °C and strain rates of 0.1 s^−1^ and 0.01 s^−1^) with those of pure copper (grain size of 20 µm) in the reference [25] reveals that the graphene/copper composites show an increase in flow stress by 10–15 MPa compared to that of pure copper. During hot deformation, graphene can hinder the further migration of dislocations and grain boundaries, which leads to an increase in the rheological stress of the composites [26,27]. Therefore, the TMP of composites is more difficult compared to pure copper. Current research has shown that graphene can increase electrical conductivity. So, it is important to select the appropriate TMP parameters.

**Figure 6:**
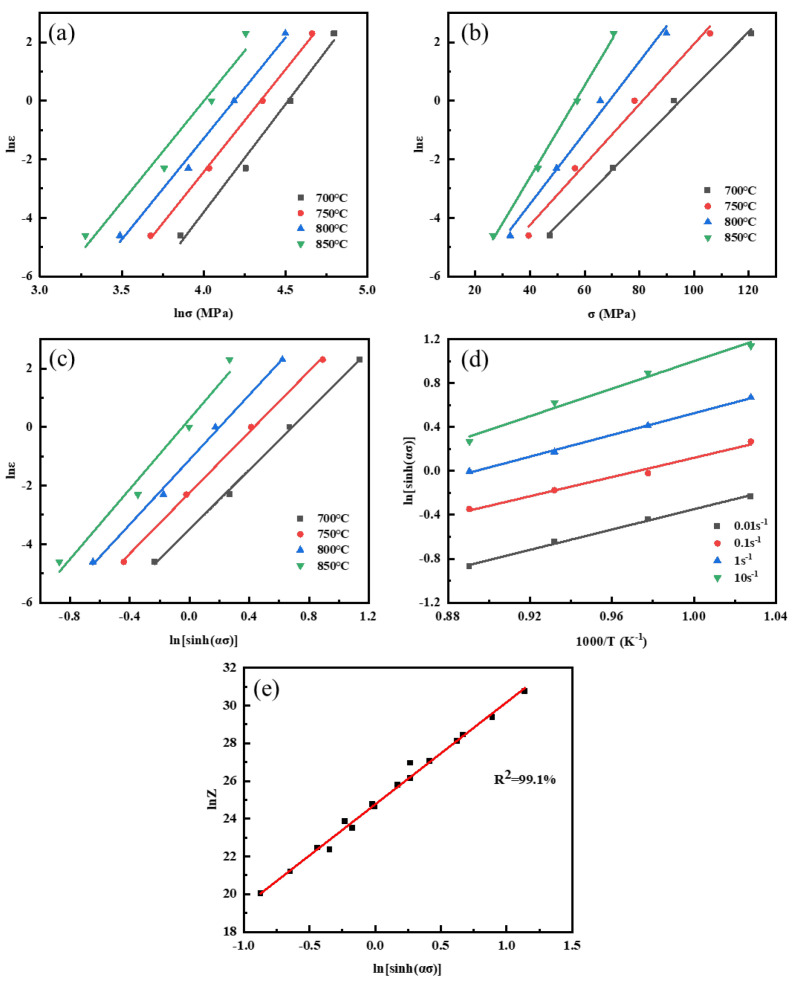
Fitting curve between TMP parameters and stress: (**a**) $\ln\dot{\varepsilon }-\ln\sigma$; (**b**) $\ln\dot{\varepsilon }$−σ; (**c**) $\ln\dot{\varepsilon }$−$\ln\left[\sin h\left(\alpha \sigma \right)\right]$; (**d**) $\ln\left[\sin h\left(\alpha \sigma \right)\right]$−$\left(1/T\right)$; (**e**) ln⁡Z−$\ln\left[\sin h\left(\alpha \sigma \right)\right]$.

The Arrhenius equation is always used to express the relevance between flow stress and TMP parameters, as follows [28,29]:

(1) $$\dot{\varepsilon }=A_{1}\sigma^{n_{1}}\exp\left(-Q/RT\right),\alpha \sigma <0.8$$

(2) $$\dot{\varepsilon }=A_{2}\exp\left(\beta \sigma \right)\exp\left(-Q/RT\right),\alpha \sigma >1.2$$

(3) $$\dot{\varepsilon }=A{\left[\sin h\left(\alpha \sigma \right)\right]}^{n}\exp\left(-Q/RT\right),\mathrm{for}\;\mathrm{all}\;\mathrm{stress}$$

(4) $$Z=A{\left[\sin h\left(\alpha \sigma \right)\right]}^{n}$$

where A_1_, A_2_, A, n_1_, n, α, and β are material constants; α = β/n_1_, R is the gas constant; *T* is the absolute temperature; Q is the thermal deformation activation energy; *Z* is the Zener-Hollomon temperature-compensated strain rate factor; *σ* is the flow stress of composites; and the peak stress is used in the solution process [30]. The logarithmic conversion of Equations (1)–(4) is obtained:

(5) $$\ln\dot{\varepsilon }=\ln A_{1}-Q/\left(RT\right)+n_{1}\ln\sigma ,\alpha \sigma <0.8$$

(6) $$\ln\dot{\varepsilon }=\ln A_{2}-Q/\left(RT\right)+\beta \sigma ,\alpha \sigma >1.2$$

(7) $$\ln\dot{\varepsilon }=\ln A-Q/\left(RT\right)+n\ln\left[\sin h\left(\alpha \sigma \right)\right]$$

(8) $$\ln Z=\ln A+n\ln\left[\sin h\left(\alpha \sigma \right)\right]$$

n_1_, β, and α are first solved. In Equations (5) and (6), n_1_ and β are the slopes of $\ln\dot{\varepsilon }-\ln\sigma$ and $\ln\dot{\varepsilon }-\sigma$, respectively. The relevance between $\ln\dot{\varepsilon }-\ln\sigma$ and $\ln\dot{\varepsilon }-\sigma$ is plotted and linearly fitted for different temperatures, as shown in Figure 6a,b. n_1_ is shown by the average of the three straight lines in the $\ln\dot{\varepsilon }-\ln\sigma$ plot with lower peak stress, and β is shown by the average slope of three straight lines in the $\ln\dot{\varepsilon }-\sigma$. It can be calculated that β = 0.10637, n_1_ = 6.92935, and α = β/n_1_ = 0.01535 mm^2^/N. n and Q can be subsequently solved. The derivation of Equation (7) is as follows:

(9) $$Q=\mathrm{Rnb}=R\cdot \frac{\partial \ln\dot{\varepsilon }}{\partial \ln\left[\sin h\left(\alpha \sigma \right)\right]}\cdot \frac{\partial \ln\left[\sin h\left(\alpha \sigma \right)\right]}{\partial \left(1/T\right)}$$

The relationship between $\ln\dot{\varepsilon }$−$\ln\left[\sin h\left(\alpha \sigma \right)\right]$ and $\ln\left[\sin h\left(\alpha \sigma \right)\right]$−$\left(1/T\right)$ is plotted and linearly fitted for different stress rates as shown in Figure 6c,d. After calculation, n = 5.44701, b = 5.05414, and Q = Rnb = 228.88390 kJ/mol. Finally, A and Z are derived. The relationship between *Z*, $\dot{\varepsilon }$, and *T* can be derived by combining Equation (8) with Equation (7):

(10) $$\ln Z=\ln\dot{\varepsilon }+Q/\left(RT\right)$$

From Equation (8), it can be seen that ln⁡A is the intercept of ln⁡Z−$\ln\left[\sin h\left(\alpha \sigma \right)\right]$. The relationship between ln⁡Z−$\ln\left[\sin h\left(\alpha \sigma \right)\right]$ is plotted and linearly fitted, as shown in Figure 6e. It can be calculated that A = e^24.61263^. The parameters of the constitutive equation of the graphene/copper composite are all solved, and the constitutive equation is as follows:

(11) $$\dot{\varepsilon }=e^{24.613}{\left[\sin h\left(0.015\sigma \right)\right]}^{5.447}\exp\left(-\frac{228.884}{RT}\right)$$

From the constitutive equation, it can be observed that the stress index n and activation energy Q of the graphene/copper composites are slightly higher than those of pure copper but much lower than those of other particle−reinforced copper matrix composites. This is because graphene is distributed along the powder and grain boundaries. The strengthening mechanism of graphene is mainly load transfer reinforcement, and graphene can only impede the excessive growth of grains but cannot prevent the recrystallization process through the Orowan effect [6,31,32]. The model’s accuracy can be judged by the linear correlation of the two parameters in Figure 6e. The linear correlation coefficient reaches 99.1%, indicating that the isomorphic equation has good accuracy.

In the dynamic material model, the hot deformation process of the material is a closed thermodynamic system, and the energy *P* input into the system is dissipated through the following two processes: the energy consumed by the plastic deformation of the material denoted as dissipation *G* and the energy consumed by the microstructure transformation of the material during plastic deformation denoted as the dissipation coefficient *J*. Therefore, the energy *P* input into the system can be expressed by the following equation:

(12) $$P=\sigma \dot{\varepsilon }=G+J=\int_{0}^{\dot{\varepsilon }}\sigma d\dot{\varepsilon }+\int_{0}^{\sigma }\dot{\varepsilon }d\sigma$$

The proportionality between the dissipation *G* and the dissipation coefficient *J* can be responded to by the stress–strain sensitivity index *m*, which is given by the following equation:

(13) $$m=\frac{\partial J}{\partial G}=\frac{\partial \left(\ln\sigma \right)}{\partial \left(\ln\dot{\varepsilon }\right)}$$

When the deformation temperature and strain are certain, the intrinsic relationship between stress and strain rate is as follows:

(14) $$\sigma =K{\dot{\varepsilon }}^{m}$$

Combining Equations (13) and (14) with Equation (12):

(15) $$J=\sigma \dot{\varepsilon }-\int_{0}^{\dot{\varepsilon }}K{\dot{\varepsilon }}^{m}d\dot{\varepsilon }=\frac{m}{m+1}\sigma \dot{\varepsilon }$$

The value of m ranges from 0 to 1. A higher value of m indicates that the material consumes more energy for microstructural transformation during plastic deformation. When *m* = 1, the material is in the ideal dissipation state and “*J*” reaches the maximum value *J_max_* = $\sigma \dot{\varepsilon }/2$. The energy dissipation factor η can respond to the percentage of energy consumed by the microstructure transformation during the material deformation process with the following equation:

(16) $$\eta =\frac{J}{J_{max}}=\frac{2m}{m+1}$$

According to Equations (13) and (16), the variation rule of η with TMP parameters can be calculated, and then the power dissipation map is plotted. In the power dissipation map, the higher value of η does not represent the better processing performance of the material, and the value of η of the material in the destabilization zone may also be very high, so it is also imperative to consider the deformation destabilization behavior of the material when selecting the material processing window. According to the Prasad criterion, $\xi (\dot{\varepsilon })$ is used as the flow destabilization criterion with the following equation:

(17) $$\xi \left(\dot{\varepsilon }\right)=\frac{\partial \ln\left(\frac{m}{m+1}\right)}{\partial \ln\dot{\varepsilon }}+m<0$$

**Figure 7:**
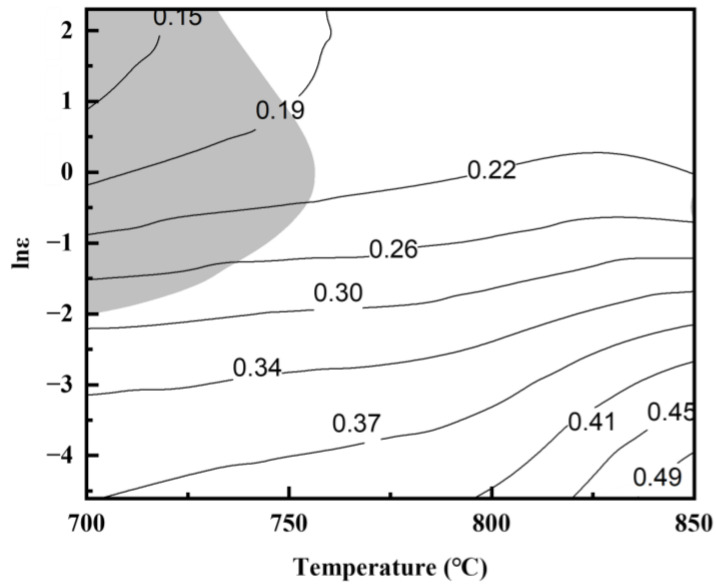
Hot processing map for graphene/copper composites.

When $\xi (\dot{\varepsilon })$ is less than zero, the material undergoes flow destabilization. According to Equations (13) and (17), the variation rule of $\xi (\dot{\varepsilon })$ with deformation temperature and strain rate can be calculated. Then, the hot processing map of graphene/Cu composites can be obtained by superimposing the above two maps, as shown in Figure 7. The white part of the map represents the safety zone, and the gray part represents the flow instability zone. As the deformation temperature increases and the strain rate decreases, the power dissipation value of composites gradually increases, indicating beneficial plastic deformation conditions. The flow instability zone of the composites is mainly concentrated in the region of low temperature and high strain rate, corresponding to TMP parameters of 700~750 °C and 0.1~10 s^−1^.

### 3.3. Microstructure after Hot Deformation

**Figure 8:**
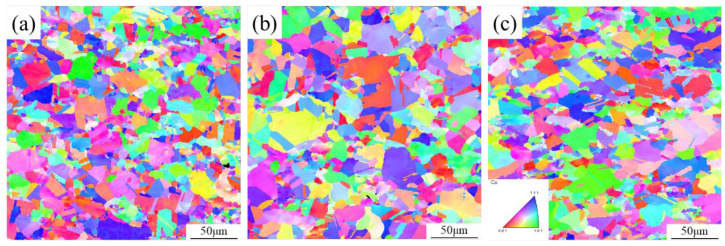
IPF maps of graphene/copper composites at different deformation temperatures: (**a**) 750 °C; (**b**) 800 °C; (**c**) 850 °C.

Firstly, samples with a strain rate of 1 s^−1^ and a deformation temperature of 750~850 °C were selected for EBSD analysis, as shown in Figure 8. The grains of graphene/copper composites at different deformation temperatures are all equiaxial with uniform size, and the grain size gradually increases from 12.7 μm to 14.4 μm with the increase in deformation temperature. Higher temperatures provide more energy for dynamic recrystallization, which increases the migration of grain boundaries, so composites have larger grains [33]. However, grains with spherical boundaries similar to the as−sintered state can also be observed in the sample deformed at 750 °C, which is due to the low temperature that cannot supply dynamic recrystallization with sufficient energy [34,35]. When the deformation temperature increases to 800 °C, composites have a moderate grain size and are located in the safety zone of the hot processing map. Further increasing the deformation temperature to 850 °C caused the slight coarsening of the grains. Therefore, 800 °C is considered a suitable TMP temperature.

**Figure 9:**
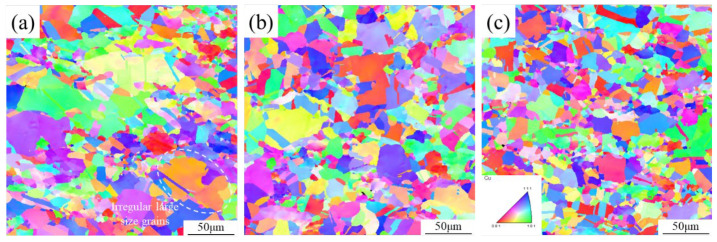
IPF diagrams of graphene/copper composites at different strain rates: (**a**) $\dot{\varepsilon }$ = 0.1 s^−1^; (**b**) $\dot{\varepsilon }$ = 1 s^−1^; (**c**) $\dot{\varepsilon }$ = 10 s^−1^.

Figure 9 shows EBSD microstructures of the graphene/copper composites with a deformation temperature of 800 °C and a strain rate of 0.1~10 s^−1^. The grain size decreases from 19.0 μm to 12.2 μm with the increase in strain rate. Moreover, some serious irregular grain growth occurs when the strain rate is 0.1 s^−1^. As mentioned above, network graphene in the composites can hinder the migration of dislocations and the growth of DRX grains. However, the network structure of graphene will be broken during the TMP procedure [13]. Due to the high temperature and the slow strain rate, dislocations have sufficient time to move and can traverse the notch of the graphene network. Thus, with the assistance of such activated dislocation motion, the DRX grains could combine adjacent sub−grains and eventually form irregular large−sized grains [34,36]. When the strain rate increases to 1 s^−1^, the deformation time becomes shorter. Dislocations have very limited time to move and the DRX grains are also restrained to grow. This caused the entanglement of dislocations and formed dense sub−grain boundaries. These sub−grains were able to transform into DRX grains during the subsequent steady flow stage. Consequently, the deformed graphene/copper composites exhibited a more homogeneous and finer microstructure than that of the as−sintered composites [24]. When the strain rate further increased to 10 s^−1^, though the DRX grains became finer, there were some initially coarse grains. Such an inhomogeneous microstructure is detrimental to mechanical and conductive performance, which needs to be avoided. Thus, the optimum TMP parameters were determined to be 800 °C and 1 s^−1^, respectively, in which a uniform microstructure with fine grains can be obtained in the graphene/copper composites.

## 4. Conclusions

In this study, the hot deformation behavior of graphene/copper composites was investigated at deformation temperatures of 700~850 °C and strain rates of 0.01~10 s^−1^. The conclusions are as follows:

(1) The hot deformation mechanism of graphene/copper composites is dynamic recrystallization (DRX), and such DRX mediated deformation behavior could be accurately described by the established Arrhenius model.

(2) The graphene/copper composite has a stress index of 5.45 and an activation energy of 228.98 kJ/mol, indicating that the energy input to obtain a fully recrystallized microstructure in graphene/copper composites is slightly higher than that in pure copper but significantly lower than that in particle-reinforced copper composites.

(3) The grain size of graphene/copper composites was sensitive to the strain rate and less affected by deformation temperature. The optimum TMP parameters were determined to be 800 °C and 1 s^−1^, respectively, at which a uniform microstructure with fine grains can be obtained.

## Data Availability

The data presented in this study are available on request from the corresponding author.
